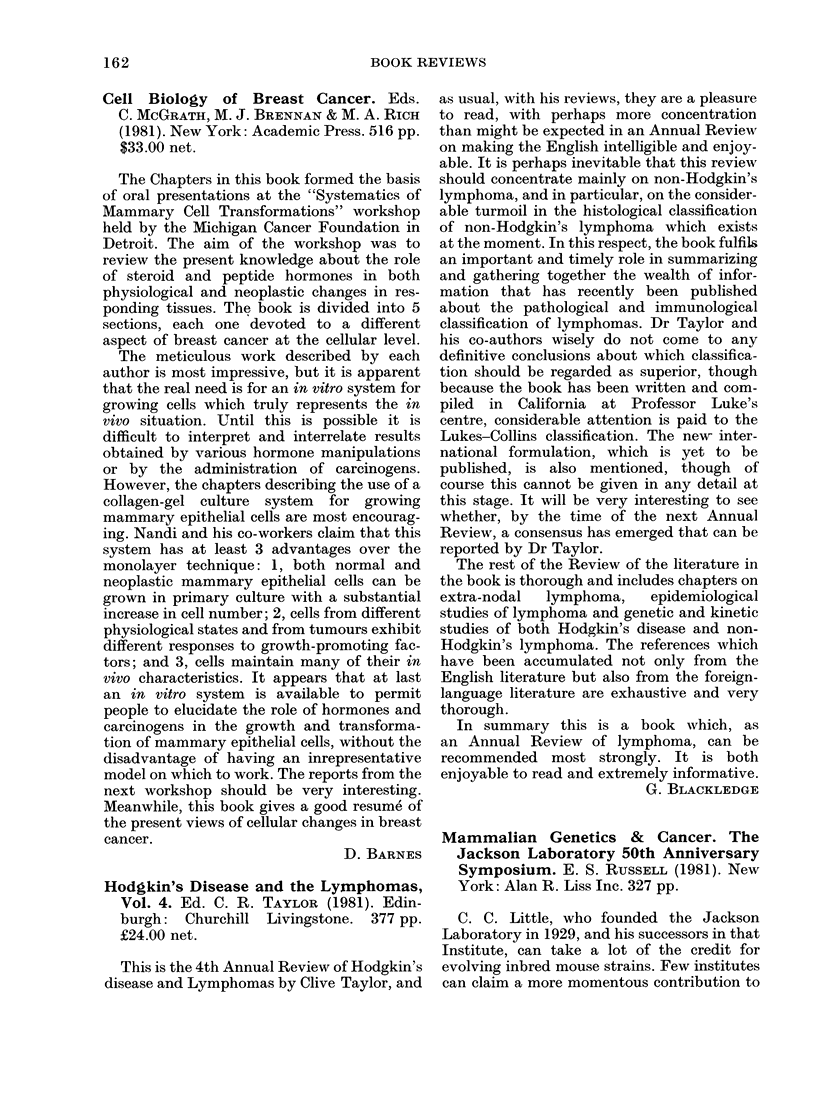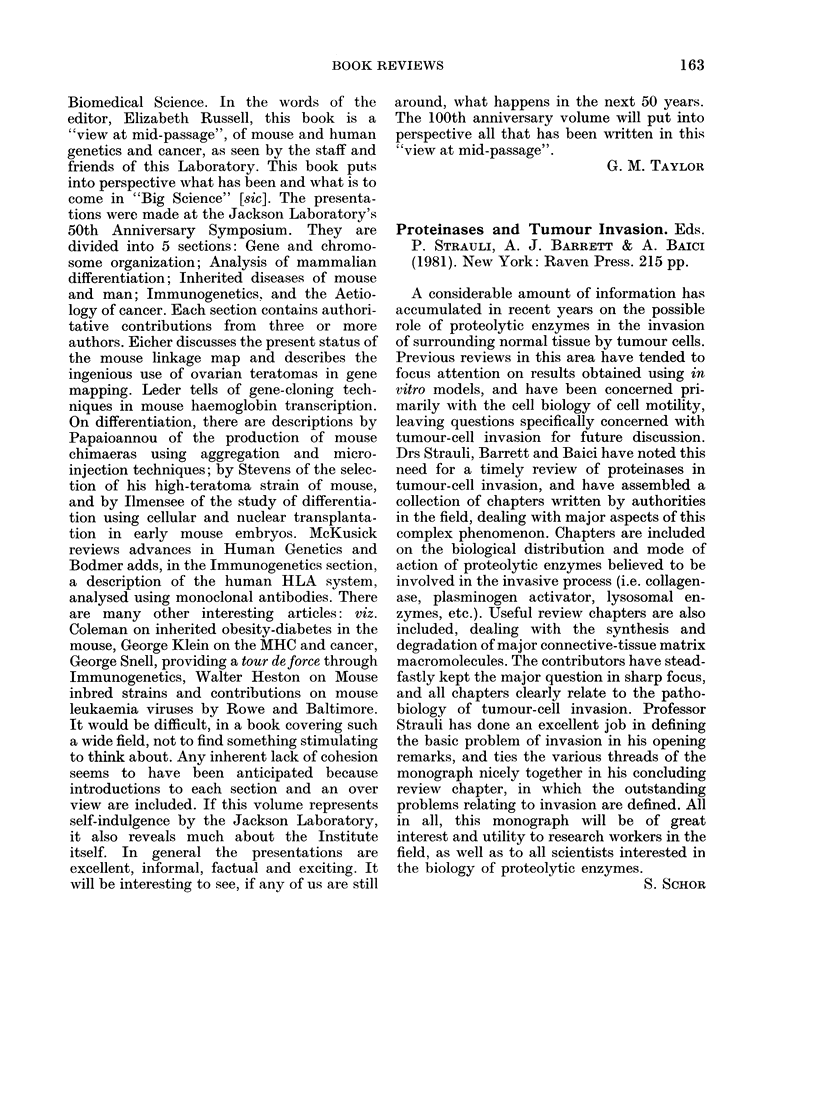# Mammalian Genetics & Cancer. The Jackson Laboratory 50th Anniversary Symposium

**Published:** 1982-01

**Authors:** G. M. Taylor


**Mammalian Genetics & Cancer. The Jackson Laboratory 50th Anniversary Symposium.** E. S. Russell (1981). New York: Alan R. Liss Inc. 327 pp.

C. C. Little, who founded the Jackson Laboratory in 1929, and his successors in that Institute, can take a lot of the credit for evolving inbred mouse strains. Few institutes can claim a more momentous contribution to

Biomedical Science. In the words of the editor, Elizabeth Russell, this book is a "view at mid-passage", of mouse and human genetics and cancer, as seen by the staff and friends of this Laboratory. This book puts into perspective what has been and what is to come in "Big Science" [*sic*]. The presentations were made at the Jackson Laboratory's 50th Anniversary Symposium. They are divided into 5 sections: Gene and chromosome organization; Analysis of mammalian differentiation; Inherited diseases of mouse and man; Immunogenetics, and the Aetiology of cancer. Each section contains authoritative contributions from three or more authors. Eicher discusses the present status of the mouse linkage map and describes the ingenious use of ovarian teratomas in gene mapping. Leder tells of gene-cloning techniques in mouse haemoglobin transcription. On differentiation, there are descriptions by Papaioannou of the production of mouse chimaeras using aggregation and microinjection techniques; by Stevens of the selection of his high-teratoma strain of mouse, and by Ilmensee of the study of differentiation using cellular and nuclear transplantation in early mouse embryos. McKusick reviews advances in Human Genetics and Bodmer adds, in the Immunogenetics section, a description of the human HLA system, analysed using monoclonal antibodies. There are many other interesting articles: *viz.* Coleman on inherited obesity-diabetes in the mouse, George Klein on the MHC and cancer, George Snell, providing a *tour de force* through Immunogenetics, Walter Heston on Mouse inbred strains and contributions on mouse leukaemia viruses by Rowe and Baltimore. It would be difficult, in a book covering such a wide field, not to find something stimulating to think about. Any inherent lack of cohesion seems to have been anticipated because introductions to each section and an over view are included. If this volume represents self-indulgence by the Jackson Laboratory, it also reveals much about the Institute itself. In general the presentations are excellent, informal, factual and exciting. It will be interesting to see, if any of us are still around, what happens in the next 50 years. The 100th anniversary volume will put into perspective all that has been written in this "view at mid-passage".

G. M. TAYLOR